# Evaluation of Five Ready-to-Use Bases for the Topical Administration of Propranolol Hydrochloride to Treat Infantile Hemangioma

**DOI:** 10.3390/pharmaceutics17010083

**Published:** 2025-01-10

**Authors:** Chiara Lacassia, Annalisa Cutrignelli, Flavia Maria la Forgia, Sergio Fontana, Antonio Lopalco, Nunzio Denora, Angela Assunta Lopedota

**Affiliations:** 1 Department of Pharmacy-Pharmaceutical Sciences, University of Bari Aldo Moro, 70125 Bari, Italy; chiara.lacassia@uniba.it (C.L.); annalisa.cutrignelli@uniba.it (A.C.); nunzio.denora@uniba.it (N.D.); angelaassunta.lopedota@uniba.it (A.A.L.); 2 Centro Studi Ricerche “Dr. S. Fontana 1900–1982”, Farmalabor s.r.l., 76012 Canosa di Puglia, Italy; f.laforgia@farmalabor.it (F.M.l.F.); s.fontana@farmalabor.it (S.F.)

**Keywords:** propranolol hydrochloride, formulation, topical, hemangioma, pediatric, compounding, off-label, repurposing, repositioning

## Abstract

**Background/Objectives**: Since 2008, following clinical studies conducted on children that revealed the ability of the β-adrenergic antagonist propranolol to inhibit capillary growth in infantile hemangiomas (IHs), its oral administration has become the first-line treatment for IHs. Although oral propranolol therapy at a dosage of 3 mg/kg/die is effective, it can cause systemic adverse reactions. This therapy is not necessarily applicable to all patients. Topical skin applications could help maintain a high drug concentration at local sites and also represent a characteristically easy method of administration for pediatric patients. Because no topical propranolol dosage forms are commercially available, such formulations may be prepared at hospitals and pharmacies. **Methods**: In the present study, we identified a simple method for preparing topical propranolol hydrochloride formulations at 1% *w/w* with five commercial ready-to-use bases and evaluated the pharmaceutical profiles. The physical stability of the extemporaneous formulations was predicted by performing an accelerated centrifuge test and assessed by visual inspection after one month storage at 25 °C. The chemical stability of the drug in the five formulations was assessed by using a high-performance liquid chromatography (HPLC) method. In vitro drug-release and permeability experiments were conducted through synthetic membranes and the outer pavilion of a pig’s ear by utilizing Franz-type diffusion cells. **Results**: The results indicated that the release of the drug was significantly influenced by the internal structure and physicochemical properties of each base. **Conclusions**: Specifically, the formulations prepared with the hydrophilic bases could be easily prepared and yield satisfactory results, representing a potential effective therapy for IHs in pediatric patients.

## 1. Introduction

Infantile hemangiomas (IHs) are the most common childhood benign tumors [1], caused by uncontrolled proliferation from vascular endothelial cells. IHs appear soon after birth, forming skin spots that may be light in hue and covered with dilated small capillaries named telangiectasias. These tumors grow rapidly in size, intensity, color (red or bluish spots), and texture and tend to stabilize or regress in 7 to 8 years [2,3]. Often, IHs do not cause significant concern and only require careful monitoring of the skin lesion. However, in about 12% of cases, especially in multiple or segmental forms, complications can arise that may require specialist consultation and prompt treatment [4].

Until 2008, local or oral administration of corticosteroids, interferon-alpha, and vincristine was the first-line treatment of choice for IHs together with highly invasive therapy, such as surgical and laser interventions. In 2008 in France, the “off-label” therapeutic benefits of oral propranolol in the benign tumor IHs were randomly discovered in a clinical trial conducted on children with hypertrophic obstructive cardiomyopathy [5]. Since then, the oral administration of propranolol [6] has become the first-line treatment for IHs, showing its ability to block the tumor’s growth phase and rapidly induce regression, leading to its repurposing for the treatment of various rare diseases [7,8].

**Figure 1:**
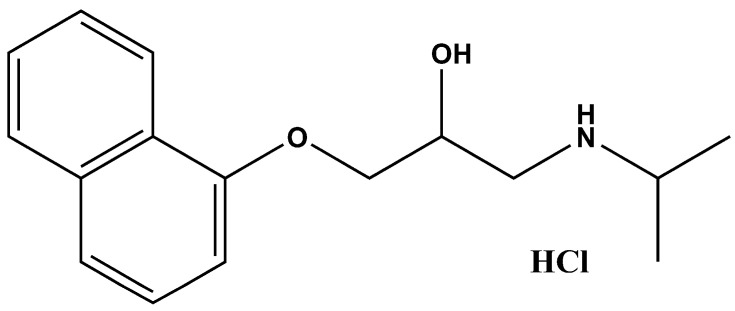
Chemical structure of propranolol hydrochloride (PRP-HCl).

Propranolol hydrochloride (PRP-HCl) (Figure 1) is a non-specific β1- and β2-adrenergic receptor (ADRB1-2) antagonist, prescribed for hypertension, irregular heart rate, essential tremor, and anxiety.

However, oral administration of PRP-HCl for the treatment of IHs can cause several adverse effects, such as bradycardia, hypotension, and hypoglycemia [9]. Therefore, topical formulations of PRP-HCl are considered an appealing approach to selectively deliver the drug at the focal sites of the tumor and easily administer the therapy to pediatric patients [6]. Nowadays, there is no industrial topical medicine of PRP-HCl, so to provide appropriate therapy for IHs, the only alternative is to prepare extemporaneous formulations in a pharmacy or hospital.

Several studies have highlighted substantial variability in the formulations of topical propranolol, employing diverse pharmaceutical vehicles. For instance, Xu et al. prepared an ointment by combining crushed propranolol tablets with petroleum jelly [10], Kunzi-Rapp compounded a hydrophilic ointment in a hospital pharmacy [11], and Price et al. reported the development of a nano-propranolol hydrogel designed to enhance transdermal delivery [12].

The preparation of topical propranolol demonstrates a high degree of customization across studies, underscoring the adaptability of pharmaceutical compounding to meet specific therapeutic requirements. However, no universally recognized optimal dosage for topical propranolol in the treatment of IHs has been established. The concentrations employed in studies ranged from 0.5% to 5% *w/w*. Notably, Price et al.’s systematic review found no correlation between higher concentrations and improved efficacy [12], suggesting that lower concentrations may be equally effective while reducing the risk of local adverse effects. Although a single optimum dosage regimen remains elusive, the evidence suggests that a PRP-HCl concentration of 1% to 2% *w/w* appears effective [13,14].

In the galenic field, vehicle preparation is the longest and most delicate phase of setting up an extemporaneous formulation. A series of small-scale tests to fine-tune the most suitable vehicle for incorporating a given active are necessary, investing considerable time and raw materials. Pharmacists can expedite extemporaneous formulation by employing ready-to-use vehicles while upholding the highest quality standards [15,16]. The composition of the ready-to-use base, however, must be carefully evaluated as it can influence the stability of the active ingredient, its release properties, and its ability to permeate through the skin, determining the effectiveness of the therapy. Therefore, a systematic study on different ready-to-use bases could be useful to identify an appropriate vehicle for a topical drug to minimize the risk of errors during compounding, leading to safer and more effective treatments for patients.

This study aimed to rationalize the choice of a ready-to-use base able to vehicle PRP-HCl for the topical treatment of IHs. In earlier investigations, a preliminary assessment was conducted to evaluate the incorporation of various active pharmaceutical ingredients (APIs), including PRP-HCl [17,18], across a broader range of ready-to-use bases already on the worldwide market. This effort aimed to provide pharmacists with optimized bases and preparation protocols that ensure the requisite quality and safety standards for galenic products. Building on these findings, five ready-to-use bases were selected as vehicles for the preparation of 1% *w*/*w* PRP-HCl formulations. Their physical stability was evaluated in real time and accelerated by centrifugation studies at 25 °C. In vitro drug-release tests were performed using Franz diffusion cells equipped with a cuprophane membrane, while for the permeability tests, the skin of the inner pinna of a pig’s ear was used. Data collected from these in vitro analyses were used to elaborate mathematical models to describe the drug-release phenomena with the best relevance. The high-performance liquid chromatography (HPLC) method demonstrated the drug’s chemical stability in the five formulations.

## 2. Materials and Methods

PRP-HCl (Lot No. F2400149), Klimt (Lot No. W2204038), Caravaggio (Lot No. W2303637), Modigliani (Lot No. W2002165), Burri (Lot No. R1818671), and Warhol (Lot No. R1811917) were provided by Farmalabor s.r.l. (Canosa di Puglia, Italy). The characteristics and compositions of the five bases are reported in Table 1: as shown in their composition, the first four bases (Burri, Caravaggio, Klimt, and Modigliani) are oil-in-water creams, while the last one (Warhol) is a hydrophobic ointment, able to emulsify a small amount of water due to the presence of lanolin and the two surfactants cetyl and stearyl alcohol.

Methanol (Lot No. M1230S, Honeywell, Charlotte, NC, USA), Water for HPLC (Lot No. 2223457), NaH_2_PO_4_ (Lot No. BCBJ8797V, Sigma-Aldrich, Milan, Italy), hydrogen peroxide (H_2_O_2_, 35%, Lot No. 21.4722903.500, CHEMLAB Analytical), NaOH pellets for analysis (Lot No. B1543269 847, Merk, Milan, Italy), H_3_PO_4_ (>85%, Lot No. SZBC 1860V, Merk), and HCl (ACS reagent 37%, Lot No. 1320A, Honeywell Fluka^TM^) were supplied by Levanchimica s.r.l. (Bari, Italy). The glass beakers, vials, graduated cylinders and pipettes, and volumetric flasks used were in borosilicate and supplied by Levanchimica s.r.l. (Bari, Italy).

### 2.1. Preparation of Topical PRP-HCl Formulations

The semisolid formulations were prepared as described in the procedures reported in the Good Preparation Practices guidelines [19,20] by solubilizing approximately 100 mg of PRP-HCl weighed on a balance in 200 µL of distilled water [17,18]. Then, the solution of the API was mixed (for 5 min) with 9.7 g of the selected base weighed on a balance in the same beaker with a glass stick until evenly dispersed and then put in a plastic pack (PP, 20 mL volume capacity, Farmalabor s.r.l.). The samples were prepared in triplicate, and the qualitative–quantitative compositions are shown in Table 2.

### 2.2. HPLC Method

Quantitative analysis of the samples was conducted by a Shimadzu HPLC Nexera series equipped with a photodiode array detector and an SIL-40C autosampler using the method described by Lopalco A. and coworkers [21]. The system was equipped with a hyperclone ODS C18 250 mm × 4.6 × 5 µm column thermostated at 35 °C. An isocratic elution at a flow rate of 1.0 mL/min was used, and the ultraviolet detection was carried out at 254 nm. Each sample injection had a 20 µL volume. The mobile phase was acetonitrile, methanol, and an aqueous solution of 0.01 M sodium phosphate dibasic adjusted to pH = 2.7 with phosphoric acid in a ratio of volumes 50:35:15 (% *v*/*v*).

A stock solution of 1 mg/mL PRP-HCl in a 30% (*v*/*v*) methanol/water mixture was used to create six different diluted solutions for the calibration curve and verify the linearity. The mean area of each sample, calculated on triplicate injections, was plotted against its concentration using the least squares regression method. The linearity range was demonstrated between 1.008 µg/mL and 100.8 µg/mL (R^2^ > 0.9999). Each analysis lasted 8 min, and the drug peak retention time was about 5 min. The slope of the calibration curve and the standard deviation of the curve response (Sy) were used to calculate the lower limit of detection (LOD) and lower limit of quantification (LOQ) by using equation 1 and 2, respectively:

(1) $$\mathrm{LOD}=3.3\frac{\mathrm{Sy}}{\mathrm{Slope}}$$

(2) $$\mathrm{LOQ}=10\frac{\mathrm{Sy}}{\mathrm{Slope}}$$

LOD and LOQ were 0.18 μg/mL and 0.54 μg/mL, respectively.

### 2.3. Accelerated and Real-Tme Physical Stability Studies

An accelerated centrifuge test was conducted to assess the formulations’ physical stability. The protocol was established starting from the evidence reported by Sopyan and coworkers [22], who reported that centrifuge tests could simulate the effect of gravity for a specific period. A total of 10 g of each sample was put in a 15 mL falcon tube and subjected to 5 centrifugation cycles of 30 min each in a ThermoScientific SL 16R centrifuge (Thermo Fisher Scientific, Rodano, Milan, Italy). The temperature and the speed were set at 25 °C and 3800 rpm, respectively. Under these conditions, one centrifugation cycle corresponded to the effect of gravity of approximately 1.2 months.

For the assessment of the real-time physical stability, each formulation was kept at 25 °C and 60% RH in a climatic chamber (Climacell; MMM Medcenter; Munich; Germany) for 30 days. The samples were observed, simulating a daily use condition, in terms of organoleptic characteristics (color and odor), pH values, and phase separation phenomena, which may serve as indicators of degradation processes or contamination over time.

### 2.4. Chemical Stability Study

The chemical stability of the API in the same samples subjected to real-time physical stability studies was investigated by HPLC analysis. Aliquots of 0.5 g of each formulation were taken and diluted in 10 mL of a 30% (*v*/*v*) methanol/water mixture, vortexed in a falcon tube, sonicated with an ultrasonic cleaner CP102 (FIOA International s.r.l., Arezzo, Italy) for two minutes, and finally centrifuged for 10 min at 25 °C and 13,000 rpm. After being separated, the supernatant was filtered with a PTFE 0.25 µm filter and diluted 10-fold with a 30% (*v*/*v*) methanol/water mixture before being analyzed by HPLC. By examining the chromatographic peaks and their areas in relation to the drug calibration curve, the concentration of PRP-HCl was determined.

### 2.5. In Vitro Release and Permeation Studies

Drug-release and permeation profiles were obtained by performing in vitro studies using Franz diffusion cells equipped with a cuprophane membrane and the skin of the inner pinna of a pig’s ear, respectively. The membrane was first hydrated with the receptor solution to facilitate interaction with the formulation. The donor compartment was filled with 200 mg of each sample (corresponding to 2 mg of API), while a 0.1 M phosphate buffer solution at pH 7.4 was placed in the receiver compartment. The system was maintained at 37 °C throughout the experiment with a circulating water bath, and air bubbles between the solution and the membrane in the receptor compartment were properly eliminated. The 500 μL samples were withdrawn from the receiver compartment at predetermined times (0, 1, 2, 3, 4, 5, 6, 24 h), replaced with fresh receiver medium, and analyzed by using the HPLC method previously described. Sample collection up to 48 h was carried out in the case of releases through a synthetic membrane. The cumulative amount permeated through the membrane was calculated from the drug concentration in the receiving medium and plotted as a function of time. The amount of drug retained in the skin was determined by extraction from the tissue incubated overnight at 4 °C in 1 mL of acetonitrile. Data are the mean of three experiments, each one conducted in duplicate.

### 2.6. Mathematical Models and Kinetic Studies

Four mathematical models, i.e., zero-order, first-order, Higuchi–Connor, and Kosmeyer–Peppas, were used to investigate the PRP-HCl kinetic release from each formulation and rationalize the involved mechanisms. The experimental data, collected before reaching equilibrium, were processed as the cumulative amount of drug released over time using the four predefined equations. The correlation coefficient (R^2^) for each mathematical model greater than 0.95 was taken into account and used as a first criterion to discard the models least suitable for describing the drug-release phenomenon. The mechanism of drug diffusion was evaluated considering the release exponent *n* calculated from the Kosmeyer–Peppas equation.

### 2.7. Statistical Analysis

Data are the mean of three experiments, each one conducted in duplicate. The relative values of the standard deviations (S.D.) were calculated using Microsoft Excel, and GraphPad Prism version 5.0 (San Diego, CA, USA) was used to fit the mathematical models in the release studies.

## 3. Results and Discussion

Currently, no commercial topical formulations of PRP-HCl are available for the treatment of IHs. To address this gap, this study aimed to evaluate five semisolid formulations prepared by using different ready-to-use bases as vehicles for the API. Ready-to-use bases in pharmaceutical compounding offer significant advantages over formulations from individual components, including greater efficiency, safety, and precision. These pre-formulated bases simplify preparation by ensuring ingredient stability and compatibility, allowing for pharmacists to focus on customizing therapies. They reduce errors, enhance stability by preventing degradation or incompatibility, and are compatible with a wide range of APIs, enabling tailored patient-specific medications without compromising formulation quality.

The five extemporaneous preparations were analyzed in terms of physical stability and in vitro drug-release and permeability profiles. The chemical stability of the drug within the formulations was also investigated, with the goal to propose a robust galenic alternative to effectively meet clinical needs.

### 3.1. Accelerated and Real-Time Physical Stability Studies

**Figure 2:**
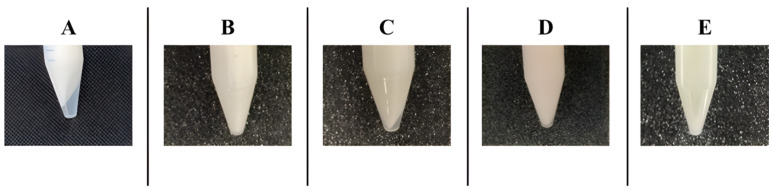
Representative images of 1% *w/w* PRP-HCl semisolid formulations in each ready-to-use base (Burri (**A**) at 4 cycles, Caravaggio (**B**) at 5 cycles, Klimt (**C**) at 4 cycles, Modigliani (**D**) at 5 cycles, Warhol (**E**) at 5 cycles) after the centrifugation test.

The parameters used in the accelerated assay, realized by a centrifugation procedure that typically exacerbates instability, simulated the effect of gravity on the sample for six months under storage conditions in order to predict phenomena such as phase separations or drug precipitation. Figure 2 shows the effect of different centrifugation cycles on each sample.

The lack of significant changes in the physical appearance of the creams prepared with the Caravaggio (B) and Modigliani (D) bases and the ointment prepared with the Warhol (E) base after five centrifugation cycles indicates the physical stability of these formulations up to 6 months (Figure 2). This result highlights strong excipient–drug interactions that balance well the overall formulation matrix, providing its structural integrity during the time. This is an important consideration for topical formulations, as physical stability is crucial for ensuring uniformity and consistency throughout the shelf-life of the product. In contrast, the creams prepared with the Burri (A) and Klimt (C) bases show an evident phase separation after four centrifugation cycles, highlighting a physical stability below 4 months. The different behavior observed among the formulations could be explained considering the composition and characteristics of the ready-to-use bases (Table 1). The Burri and Klimt bases are characterized by a lower viscosity (below 40,000 cPoise) compared to the Caravaggio and Modigliani bases (viscosity up to 100,000 cPoise). The viscosity of a dispersed system, such as a cream, influences its physical stability: as known from Stokes’ law, the higher the viscosity the lower the speed with which phase separation phenomena (oil and water) occur. In regards to the formulation prepared with the Warhol base, its physical stability could be explained considering several aspects: it is a lipophilic ointment in a single phase constituted prevalently by petrolatum in which the quantity of water (200 μL) necessary to solubilize PRP-HCl is well dispersed for the presence of the excipient’s lanolin, which has self-emulsifying properties, and the two surfactants cetyl and stearyl alcohol.

The real-time stability assay confirms the results observed with the accelerated test. All the formulations were stable after one month in a climatic chamber as described above. Furthermore, since the test was conducted by simulating in-use conditions of the products, the absence of variations in pH, odor, color, phase integrity, and microbiological contaminants observable by visual inspection may also indicate microbiological stability of the preparations. The lack of pH variations suggests that the formulations are well buffered and resilient to environmental factors that could otherwise cause acid–base shifts, affecting both the stability of the API and the overall product performance.

### 3.2. Chemical Stability Study

The chemical stability test represents a fundamental aspect of ensuring product quality and therapeutic efficacy. The chemical stability of PRP-HCl was demonstrated by HPLC at predefined time points (0, 15, 30 days) to quantitatively monitor the API in the formulations. Residual drug content values in the formulations stored at 25 °C are reported in Table 3. The results show that no drug degradation occurs after 30 days, since the HPLC chromatograms analyzed at each time did not highlight the presence of degradants. These findings show the suitability of the selected bases for maintaining the integrity of PRP-HCl in semisolid topical applications, providing a foundation for further in vitro investigations and clinical studies.

However, it is important to underline that, in the case of the formulation prepared with the ready-to-use Warhol base, the extracted quantity of API is always around 90%. These results can be explained by considering the nature of the base used, which is a single-phase lipophilic ointment. This type of vehicle probably has a high affinity towards the drug that is retained in the formulation and not completely extracted. In fact, PRP-HCl is a lipophilic molecule characterized by an octanol/water partition coefficient (Log P) equal to 2.81 [23]. This hypothesis will be further confirmed by the results obtained in the release studies with Franz cells.

### 3.3. In Vitro Release and Permeation Studies

**Figure 3:**
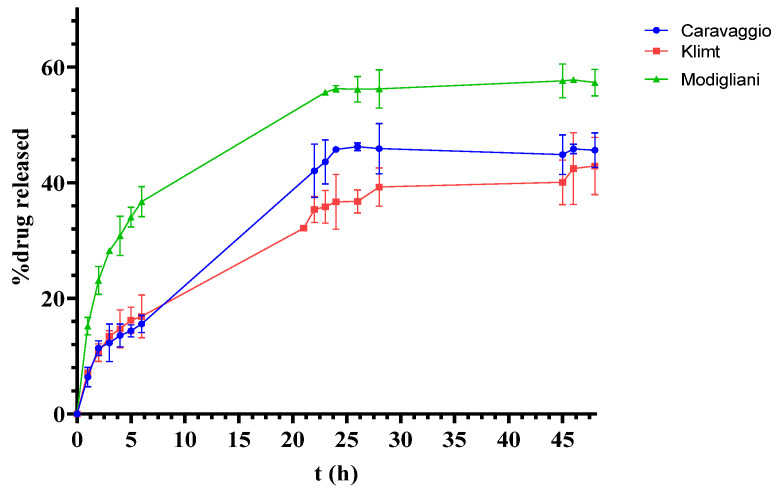
PRP-HCl release profile by Franz cell equipped with cuprophane membrane.

Figure 3 illustrates the release profiles of PRP-HCl from creams formulated with the Caravaggio, Modigliani, and Klimt bases. The data obtained with the Burri and Warhol bases are not shown because it was not possible to quantify the drug in the receptor chamber due to its inadequate release from the formulations. As underlined previously in the chemical stability, the active ingredient has a Log P value that denotes its affinity towards the Warhol lipophilic base, resulting in a poor ability to pass into the aqueous receiving medium. Instead, in the case of the Burri base, the parameter limiting the drug release could be represented by its pH value (~3.5, see Table 1), lower than that of the other creams. Since the API is a hydrochloride salt, at that pH value, it is probably present in a dispersed form that is not able to cross the cuprophane membrane.

The Franz cell drug-release study revealed differences in the profiles among the other three tested formulations over 48 h. The Modigliani base exhibited the highest release percentage, with 57.61% ± 1.33 of PRP-HCl diffused through the membrane, followed by the Caravaggio base at 39.63% ± 1.73 and the Klimt base at 37.89% ± 2.84. These results suggest that the composition of the Modigliani base may enhance the solubilization or diffusion of PRP-HCl, potentially due to optimized interactions between the drug and the excipients. In details, as shown in Table 1, disodium EDTA, a well-known chelating agent, is present in the Modigliani base. EDTA can chelate metal ions, which are sometimes involved in the formation of insoluble salts or complexes with certain drugs. By binding these ions, EDTA could increase the solubility of the drug, enhancing its release from the formulation. In fact, the Caravaggio base, which has no EDTA in its composition, shows a lower drug-release value. On the other hand, the Klimt base, although containing EDTA, has a lower petrolatum content in its composition, partly replaced by liquid petrolatum (Table 1). Liquid petrolatum could likely create a real barrier on the hydrophilic cuprophane membrane, preventing the drug from crossing it.

Further investigation into the rheological properties and microstructural characteristics of these formulations may provide insights into the factors influencing release performance.

These findings emphasize the critical role of the base composition in determining the release kinetics of PRP-HCl and highlight the importance of tailoring formulation properties to achieve the desired therapeutic outcomes.

**Figure 4:**
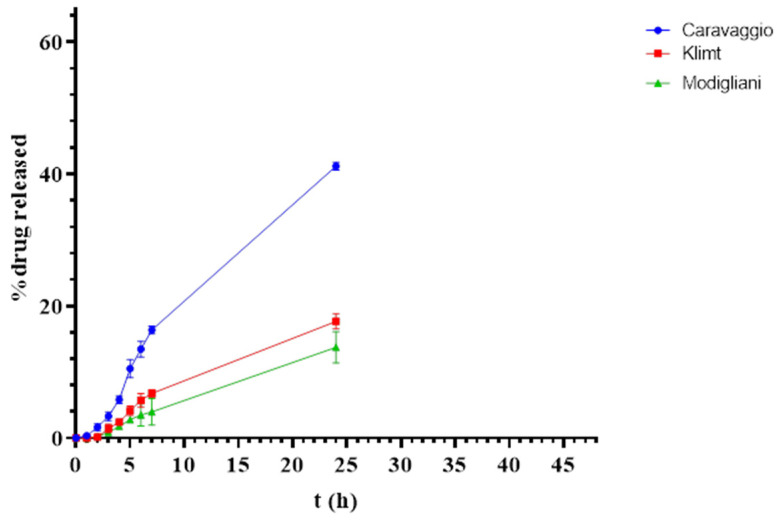
PRP-HCl release profile by Franz cell equipped with pig ear inner pavilion membrane.

In contrast, the permeability study using the skin of the inner pinna of a pig’s ear as the membrane demonstrated a markedly different trend in the percentage of PRP-HCl permeated over 24 h, specifically 13.75% ± 1.35, 17.68% ± 0.65, and 41.22% ± 0.35 for the Modigliani, Klimt, and Caravaggio bases, respectively (Figure 4). These findings suggest that, while the Modigliani base exhibited the highest release rate in the Franz cell studies with the cuprophane membrane, its permeation across a biological membrane was significantly lower compared to the Caravaggio base. This discrepancy may be attributed to the differing structural and chemical characteristics of the membranes. The cuprophane membrane, being synthetic and relatively inert, primarily reflects the release capacity of the formulation, whereas the pig’s ear membrane, which better mimics human skin, provides a more physiologically relevant barrier, where factors such as lipid compatibility, excipient–drug interactions, and formulation viscosity could play a critical role in drug permeation.

It is important to underline that, in the permeability study, the intact skin of a pig’s ear was used without separating the epidermis from the dermis. It is, therefore, a multilayer barrier with a lipophilic (stratum corneum) and hydrophilic (living epidermis and dermis) character in which the skin appendages are also present. Therefore, it is difficult to rationalize the result obtained by simply considering the compositions of the three ready-to-use bases. The superior permeation performance of the Caravaggio base (41.22%) across the pig’s ear skin compared to the Modigliani (13.75%) and Klimt (17.68%) bases suggests that its composition may enhance drug partitioning into and diffusion through the lipid-rich environment of the biological membrane.

This highlights the importance of integrating both release and permeability studies when evaluating topical formulations. While synthetic membranes provide valuable insights into release profiles, biological membranes are essential for assessing real-world drug-delivery potential. Further characterization of the bases, including their lipid compatibility, partition coefficients, and interaction with skin components, will be necessary to fully elucidate the mechanisms driving these observed differences.

In addition to drug permeation, the amount of PRP-HCl accumulated in the pig’s ear skin over 24 h was also evaluated, revealing values of 7.94% ± 0.3, 5.18% ± 0.23, and 4.45% ± 0.19 for the Modigliani, Caravaggio, and Klimt bases, respectively. These results are fully in agreement with the release and permeability data. In fact, the Modigliani base, which is the one that allows for a greater release of PRP-HCl, showed a lower permeability due to a lower ability to promote drug diffusion through the skin, guaranteeing a greater accumulation therein. This suggests that the Modigliani formulation may promote stronger interaction or binding of the drug to components of the biological membrane, such as lipids or proteins, leading to higher accumulation. Such retention could be advantageous for formulations designed for localized action, as it may prolong the presence of the drug at the site of application and potentially enhance therapeutic efficacy.

Conversely, the Caravaggio and Klimt bases exhibited lower drug accumulation in the skin, having higher drug permeation compared to the Modigliani base, favoring systemic absorption rather than localized retention. This behavior could decrease the therapeutic efficacy of the topical treatment, leading to the potential appearance of side effects. Furthermore, the possibility to have the lowest PRP-HCl permeated amounts seems interesting for a topical preparation intended to treat newborn patients. The limited drug permeation profile of the Modigliani base compared to that of the Caravaggio and Klimt creams evaluated on the pig ear inner pavilion membrane can be advantageous in such patients because their skin barrier is more permeable to xenobiotics in comparison to adults or children older than three-years old [13,14]. These observations underline the importance of balancing drug retention and permeation based on the therapeutic goals of the formulation. For conditions requiring localized drug delivery, such as in the treatment of IHs, cream formulations, like that obtained with the Modigliani ready-to-use base, may be preferable due to their enhanced accumulation within the dermis, where capillaries are localized.

### 3.4. Mathematical Models and Kinetic Studies

For the analysis of release profiles (Figure 3), in order to obtain valuable insights into the underlying mechanisms governing drug release from the different formulations [24], the most widespread kinetic models that best describe the release from semisolid systems are taken into consideration, i.e., the zero-order model, the first-order model, and the Higuchi–Connors model (Table 4). As described in Section 2.6, the correlation coefficient (R^2^) for each mathematical model greater than 0.95 was taken into account and used as a first criterion to discard the models least suitable for describing the drug-release phenomenon. In a second moment, once the kinetic model that best fit the experimental data was identified, the mechanism of drug diffusion was evaluated considering the release exponent *n* calculated from the Kosmeyer–Peppas equation, as described in the literature [25].

This was further supported by the release exponent *n*, calculated by the Korsmeyer–Peppas equation, being less than 0.5, suggesting that the drug release follows a diffusion-controlled process, where the rate is primarily governed by the concentration gradient and the drug diffusion through the matrix of the base. The Higuchi–Connors model is commonly observed in systems where the drug is released from a homogeneous matrix, and its release is not significantly hindered by the base itself.

In contrast, the Caravaggio base exhibited a different release profile, following first-order kinetics with an anomalous drug transport mechanism. The release exponent *n* for this formulation was greater than 0.5, indicating a non-Fickian or anomalous transport process (Table 4). This suggests that the release from the Caravaggio base may involve complex mechanisms, such as drug diffusion coupled with matrix relaxation or swelling, which could result in a release rate that is not solely dependent on the drug concentration gradient. The first-order behavior is often seen in systems where the drug release is controlled by both diffusion and the dissolution of the base, leading to a more sustained release over time.

These findings underscore the significant role that the vehicles, such as the ready-to-use bases, play in determining the release kinetics of the drug. The Modigliani and Klimt bases, which favor Fickian diffusion, may be more suitable for controlled-release systems, where a predictable and linear release is desired. On the other hand, the Caravaggio base, with its anomalous transport mechanism, could be advantageous for applications requiring more complex or sustained drug release, where the rate of release is not strictly linear.

Overall, this study highlights the importance of carefully selecting and optimizing formulation bases to tailor the release profile to meet specific therapeutic needs. Understanding the underlying release mechanisms, as revealed by the kinetic modelling, is crucial for designing more effective drug delivery systems that can achieve desired pharmacokinetic profiles.

## 4. Conclusions

Since 2008, propranolol has gained approval as the first-line treatment for IHs associated with risks of dysfunction and esthetic problems due to its high effectiveness. Following the success of oral treatment, topical formulations of the API have been explored, particularly for curing superficial IHs in pediatric patients who may be more prone to oral treatment side effects. In fact, topical skin applications could help maintain high drug concentrations at local or focal sites and also represent an easy method of administration to pediatric patients. Because no topical propranolol formulations are commercially available, such dosage forms may be prepared as extemporaneous formulations at hospitals and pharmacies. In the present study, we identified a simple method for preparing topical PRP-HCl formulations at 1% *w/w* with four hydrophilic creams and one lipophilic ointment commercially available. The five bases guaranteed physical stability of the dosage form and chemical stability of the API when the formulations were stored at 25 °C for one month. An in vitro drug-release screening showed that the Modigliani, Caravaggio, and Klimt hydrophilic creams could be identified as the most suitable candidates for the preparation of topical formulations. Further in vitro permeation studies highlighted the most critical formulative aspects for dermal delivery of PRP-HCl and allowed for the rational selection of the most appropriate cream. In particular, the Modigliani base ensured both higher drug release from the vehicle and higher drug concentration in the skin. Moreover, the in vitro results on full-thickness skin suggested a lower permeation pattern that could suggest lower systemic side effects induced by topical propranolol rather than oral treatment. The overall results discussed in this work may be, therefore, useful for physicians for driving the selection of a semisolid matrix according to the therapeutic needs of pediatric patients. Additionally, this systematic study allowed for the identification of an appropriate ready-to-use vehicle for a topical treatment of IHs in order to minimize the risk of errors that a pharmacist could make during compounding. By addressing critical aspects of safety, stability, and customization while reducing preparation time, these bases enable pharmacists to deliver high-quality, patient-specific medications with greater confidence and precision. Topical formulations of PRP-HCl for the treatment of IHs offer a safer and less toxic alternative to oral administration. By applying the drug directly to the affected area, the dose required is significantly lower, further decreasing the potential for systemic toxicity. However, it is essential to monitor the skin’s response and the potential for local irritation or other mild side effects, although these are generally rare and less severe than the systemic effects associated with oral propranolol therapy.

## Figures and Tables

**Table 1:** Characteristics and composition of ready-to-use bases.

Base	pH and Viscosity (cP)	Composition
**Burri** Cream base	3.5 ± 0.5; 4000–30,000	Aqua q.s. to 100%; Glyceryl stearate SE 8%; Cetyl Alcohol/Stearyl Alcohol 2%; Oryza sativa brain oil 5%; Ethylhexyl Palmitate 7%; Tocopheryl acetate 0.25%; Sodium Cetearyl sulfate 1.5%; Glycerin 2%; Benzyl alcohol 0.5%; Xanthan gum 0.3%; Lecithin 0.2; Tocopherol 0.2%; Ascorbyl Palmitate 0.1; Citric Acid 0.05%; Tartaric acid q.s. to the required pH.
**Caravaggio** Amphiphilic cream base	5.5 ± 0.5; 20,000–100,000	Aqua q.s. to 100%; Petrolatum 25.5%; Propylene Glycol 10%; Caprilyc/Capric Triglyceride 7.5%; PEG-40 stearate 7%; Cetearyl Alcohol 6%; GlycerylStearate 4%; Phenoxyethanol 0.5%; Sodium Benzoate 0.4%; Potassium Sorbate 0.3%; Citric Acid q.s. to the required pH.
**Klimt** Cetomacrogol cream base	5.5 ± 0.5; 4000–40,000	Aqua q.s. to 100%; Petrolatum 15%; Cetearyl Alcohol 50/50 7.2%; Paraffinum Liquidum 6%; Ceteareth-20 1.8%; Benzyl Alcohol 0.5%; Sodium Benzoate 0.4%; Potassium Sorbate 0.3%; Disodium EDTA 0.1%; Citric Acid q.s. to the required pH.
**Modigliani** Antioxidant cream base	5.5 ± 0.5; 20,000–100,000	Aqua q.s.to 100%; Petrolatum 25.5%; Propylene Glycol 10%; Caprilyc/Capric Triglyceride 7.5%; PEG-40 stearate 7%; Glyceryl Stearate 4%; Cetyl Alcohol 3%; Stearyl alcohol 3%; Phenoxyethanol 0.5%; Sodium Benzoate 0.4%; Sodium Metabisulfite 0.4%; Potassium Sorbate 0.3%; Disodium EDTA 0.1%; Citric Acid q.s. to pH.
**Warhol** Petrolatum-lanolin base	-	Petrolatum q.b. a 100%; Lanolin 5%; Cetyl alcohol 2.5%; Stearyl alcohol 2.5%.

**Table 2:** Samples prepared and their qualitative–quantitative composition.

**Sample**	**Base (g)**	**API (mg)**	**Solvent (** **µL)**
Modigliani 1	9.745	100.01	200
Modigliani 2	9.777	100.20	200
Modigliani 3	9.721	100.39	200

**Sample**	**Base (g)**	**API (mg)**	**Solvent (** **µL)**
Caravaggio 1	9.746	100.84	200
Caravaggio 2	9.669	100.61	200
Caravaggio 3	9.676	101.18	200

**Sample**	**Base (g)**	**API (mg)**	**Solvent (** **µL)**
Burri 1	9.744	98.50	200
Burri 2	9.745	99.95	200
Burri 3	9.770	98.55	200

**Sample**	**Base (g)**	**API (mg)**	**Solvent (** **µL)**
Klimt 1	9.681	98.90	200
Klimt 2	9.696	98.29	200
Klimt 3	9.736	98.86	200

**Sample**	**Base (g)**	**API (mg)**	**Solvent (** **µL)**
Warhol 1	9.704	99.34	200
Warhol 2	9.776	99.40	200
Warhol 3	9.774	99.95	200

**Table 3:** Residual concentrations (%) of PRP-HCl and standard deviations (S.D.) in each sample stored at 25 °C for 30 days. Data are the mean of three experiments, each one conducted in duplicate.

Sample	PRP-HCl (%)		
	0	15 Days	30 Days
**Modigliani**	95.41 ± 3.81	95.06 ± 3.98	100.78 ± 2.58
**Caravaggio**	97.13 ± 1.22	96.96 ± 1.5	98.43 ± 5.41
**Burri**	99.37 ± 1.14	94.50 ± 6.6	99.64 ± 2.97
**Klimt**	96.62 ± 4.16	96.67 ± 1.5	97.13 ± 2.2
**Warhol**	88.74 ± 6.17	87.10 ± 4.12	90.74 ± 5.2

**Table 4:** Mathematical models of PRP-HCl release kinetics from the Modigliani, Klimt, and Caravaggio ready-to-use bases by Franz cell equipped with cuprophane membrane.

Mathematical Model		Modigliani	Klimt	Caravaggio
**Zero Order**	**Equation**	y = 2.0466x + 16.08	y = 1.2196x + 6.2199	y = 1.7843x + 6.1141
	**R^2^**	0.8575	0.9425	0.9716
**First Order**	**Equation**	y = −0.0149x + 1.9304	y = −0.0067x + 1.9741	y = −0.0109x + 1.9785
	**R^2^**	0.9432	0.9655	0.987
**Higuchi—** **Connors**	**Equation**	y = 12.178x + 3.5964	y = 6.9394x − 0.5024	y = 9.8998x − 3.1504
	**R^2^**	0.9858	0.9907	0.9711
**Kosmeyer –** **Peppas**	**Equation**	y = 0.4419x + 1.193	y = 0.4978x + 0.8261	y = 0.5653x + 0.8607
	**R^2^**	0.9829	0.9815	0.9612
	***n***	0.4419	0.4978	0.5653

## Data Availability

Data are contained within the article.

## References

[1] Hoeger P.H., Harper J.I., Baselga E., Bonnet D., Boon L.M., Atti M.C.D., El Hachem M., Oranje A.P., Rubin A.T., Weibel L. (2015). Treatment of Infantile Haemangiomas: Recommendations of a European Expert Group. Eur. J. Pediatr..

[2] Léauté-Labrèze C., Harper J.I., Hoeger P.H. (2017). Infantile Haemangioma. Lancet.

[3] Holland K.E., Drolet B.A. (2010). Infantile Hemangioma. Pediatr. Clin. N. Am..

[4] Rodríguez Bandera A.I., Sebaratnam D.F., Wargon O., Wong L.C.F. (2021). Infantile Hemangioma. Part 1: Epidemiology, Pathogenesis, Clinical Presentation and Assessment. J. Am. Acad. Dermatol..

[5] Léauté-Labrèze C., de la Roque E.D., Hubiche T., Boralevi F., Thambo J.-B., Taïeb A. (2008). Propranolol for Severe Hemangiomas of Infancy. N. Engl. J. Med..

[6] Chen Z.-Y., Wang Q.-N., Zhu Y.-H., Zhou L.-Y., Xu T., He Z.-Y., Yang Y. (2019). Progress in the Treatment of Infantile Hemangioma. Ann. Transl. Med..

[7] Cuesta A.M., Gallardo-Vara E., Casado-Vela J., Recio-Poveda L., Botella L.-M., Albiñana V. (2022). The Role of Propranolol as a Repurposed Drug in Rare Vascular Diseases. Int. J. Mol. Sci..

[8] Wassef M., Blei F., Adams D., Alomari A., Baselga E., Berenstein A., Burrows P., Frieden I.J., Garzon M.C., Lopez-Gutierrez J.-C. (2015). Vascular Anomalies Classification: Recommendations From the International Society for the Study of Vascular Anomalies. Pediatrics.

[9] Kwon E.-K.M., Joachim S., Siegel D.H., Drolet B.A., Holland K. (2013). Retrospective Review of Adverse Effects From Propranolol in Infants. JAMA Dermatol..

[10] Xu G., Lv R., Zhao Z., Huo R. (2012). Topical Propranolol for Treatment of Superficial Infantile Hemangiomas. J. Am. Acad. Dermatol..

[11] Kunzi-Rapp K. (2012). Topical Propranolol Therapy for Infantile Hemangiomas. Pediatr. Dermatol..

[12] Price A., Rai S., Mcleod R.W.J., Birchall J.C., Elhassan H.A. (2018). Topical Propranolol for Infantile Haemangiomas: A Systematic Review. J. Eur. Acad. Dermatol. Venereol..

[13] Piraccini B.M., Alessandrini A., Dika E., Starace M., Patrizi A., Neri I. (2016). Topical Propranolol 1% Cream for Pyogenic Granulomas of the Nail: Open-label Study in 10 Patients. J. Eur. Acad. Dermatol. Venereol..

[14] Casiraghi A., Musazzi U.M., Rocco P., Franzè S., Minghetti P. (2016). Topical Treatment of Infantile Haemangiomas: A Comparative Study on the Selection of a Semi-Solid Vehicle. Ski. Pharmacol. Physiol..

[15] Spennacchio A., Lopalco A., Racaniello G.F., Cutrignelli A., la Forgia F.M., Fontana S., Cristofori F., Francavilla R., Lopedota A.A., Denora N. (2024). Mucoadhesive Budesonide Solution for the Treatment of Pediatric Eosinophilic Esophagitis. Pharmaceuticals.

[16] Lacassia C., Spennacchio A., Lopedota A.A., Denora N., Lopalco A., Maria La Forgia F., Fontana S. (2024). Physical-Chemical Stability of the Extemporaneous Paracetamol Oral Suspension in Puccini Base. Int. J. Pharm. Compd..

[17] Lacassia C., Spennacchio A., Lopalco A., Lopedota A.A., la Forgia F., Fontana S., Franco M., Denora N. (2024). Lipophilic Ready to Use Vehicles Compounded Topical Medications. Int. J. Pharm. Compd..

[18] Spennacchio A., Lacassia C., Lopalco A., Lopedota A.A., la Forgia F., Fontana S., Franco M., Denora N. (2024). Hydrophilic Ready-to-Use Vehicles and Compounded Topical Medications. Int. J. Pharm. Compd..

[19] Procedure per Unguenti Secondo Le Norme Di Buona Preparazione. https://www.sifap.org/procedure-norme-di-buona-preparazione/procedure-unguenti.

[20] Procedure per Creme Secondo Le Norme Di Buona Preparazione. https://www.sifap.org/procedure-norme-di-buona-preparazione/procedure-creme.

[21] Lopalco A., Denora N., Laquintana V., Cutrignelli A., Franco M., Robota M., Hauschildt N., Mondelli F., Arduino I., Lopedota A. (2020). Taste Masking of Propranolol Hydrochloride by Microbeads of EUDRAGIT^®^ E PO Obtained with Prilling Technique for Paediatric Oral Administration. Int. J. Pharm..

[22] Sopyan I., Gozali D., Tiassetiana S. (2017). Formulation of Tomato Extracts (*Solanum lycopersicum* L.) as a Sunscreen Lotion. Natl. J. Physiol. Pharm. Pharmacol..

[23] SCHEDA DATI DI SICUREZZA PROPRANOLOLO CLORIDRATO. https://materie-prime.farmalabor.it/sds_completa/01907_it.pdf.

[24] Racaniello G.F., Balenzano G., Arduino I., Iacobazzi R.M., Lopalco A., Lopedota A.A., Sigurdsson H.H., Denora N. (2024). Chitosan and Anionic Solubility Enhancer Sulfobutylether-β-Cyclodextrin-Based Nanoparticles as Dexamethasone Ophthalmic Delivery System for Anti-Inflammatory Therapy. Pharmaceutics.

[25] Bialik-Wąs K., Miastkowska M., Sapuła P., Sycz A., Pluta K., Malina D., Chwastowski J. (2024). Kinetic Analysis of in Vitro Release Profiles of Salicylic Acid and Fluocinolone Acetonide from Dual Delivery Systems Composed of Polymeric Nanocarriers and a Hydrogel Matrix. J. Drug Deliv. Sci. Technol..

